# Systematic characterization of photoperiodic gene expression patterns reveals diverse seasonal transcriptional systems in *Arabidopsis*

**DOI:** 10.1371/journal.pbio.3002283

**Published:** 2023-09-12

**Authors:** Chun Chung Leung, Daniel A. Tarté, Lilijana S. Oliver, Qingqing Wang, Joshua M. Gendron

**Affiliations:** Department of Molecular, Cellular, and Developmental Biology, Yale University, New Haven, Connecticut, United States of America; University of California San Diego, UNITED STATES

## Abstract

Photoperiod is an annual cue measured by biological systems to align growth and reproduction with the seasons. In plants, photoperiodic flowering has been intensively studied for over 100 years, but we lack a complete picture of the transcriptional networks and cellular processes that are photoperiodic. We performed a transcriptomics experiment on *Arabidopsis* plants grown in 3 different photoperiods and found that thousands of genes show photoperiodic alteration in gene expression. Gene clustering, daily expression integral calculations, and *cis*-element analysis then separate photoperiodic genes into co-expression subgroups that display 19 diverse seasonal expression patterns, opening the possibility that many photoperiod measurement systems work in parallel in *Arabidopsis*. Then, functional enrichment analysis predicts co-expression of important cellular pathways. To test these predictions, we generated a comprehensive catalog of genes in the phenylpropanoid biosynthesis pathway, overlaid gene expression data, and demonstrated that photoperiod intersects with 2 major phenylpropanoid pathways differentially, controlling flavonoids but not lignin. Finally, we describe the development of a new app that visualizes photoperiod transcriptomic data for the wider community.

## Abstract 2

Daylength guides the timing of biological processes in plants, but a comprehensive view of the underlying transcriptional regulation is lacking. This study uses time course RNA-seq to thoroughly examine gene expression of Arabidopsis plants in three photoperiods, uncovering previously unexplored photoperiodic gene regulation.

## Introduction

Photoperiod, or daylength, is a robust seasonal cue that is measured by organisms ranging from algae [1] and fungi [2], to higher plants [3] and vertebrates [4]. This circannual signal allows the anticipation of environmental changes and thus the coordination of long-term developmental and reproductive processes, such as tuberization in potatoes [5] and maturation of animal gonads [6]. Lengthening or shortening of photoperiod beyond a normal range seen in a 24-h day can cause a distinct stress response in plants [7,8]. In humans, photoperiod influences mood variation and related conditions like seasonal affective disorder [9].

Plants have proved an influential study system for photoperiodism, mainly because the control of flowering time by photoperiod provides a readily observable and quantifiable phenotype. Photoperiodic flowering in many higher plants is regulated by the circadian clock-controlled expression of the *CONSTANS* (*CO*) gene [10]. In *Arabidopsis thaliana*, accumulation of *CO* mRNA occurs in late afternoon—a time that is lit only during the long photoperiods of summertime. Therefore, only in long photoperiods can the CO protein be stabilized by light and trigger the downstream inducers of flowering, namely *FLOWERING LOCUS T* (*FT*). This overlap between photoperiod and the rhythmic expression of *CO* thus defines the external coincidence mechanism. Transcriptionally, CO is proposed to control a small number of genes directly yet maintains a large indirect effect on gene expression and development by triggering the developmental switch from vegetative growth to flowering [11–13].

Growth is also under the control of photoperiod in plants, and recently, 2 photoperiod-measuring mechanisms that support or promote photoperiodic growth have been discovered. Photoperiodic control of hypocotyl elongation by phytochrome-interacting factors (PIFs) relies on a coincidence mechanism, similar to the CO-FT regulon, although PIFs have a wide variety of functions apart from regulating genes in a photoperiodic manner [14]. The circadian clock phases the expression of *PIF4/5* to the morning and late night, but the PIF4/5 proteins are only stabilized in the dark and thus promote nighttime expression of growth-regulating genes, namely *ARABIDOPSIS THALIANA HOMEOBOX PROTEIN 2* (*ATHB2*) [15–18]. Therefore, PIF4/5-regulated hypocotyl elongation occurs in the latter portion of the long night during short-day photoperiods.

Recently, a metabolic daylength measurement (MDLM) system was shown to support rosette fresh weight generation in long days and short days [19–22]. This system relies on the photoperiodic control of sucrose and starch allocation in order to control expression of the genes *PHLOEM PROTEIN 2-A13* (*PP2-A13*) [21] and *MYO-INOSTOL-1 PHOSPHATE SYNTHASE 1* (*MIPS1*) [22], which are required to support short- and long-day vegetative growth, respectively. Like the CO-FT and PIF regulatory modules, the MDLM system requires a functional circadian clock for photoperiod measurement, although the molecular connections between the clock and metabolism for this system have not been identified. Additionally, both the transcription factor(s) that control MDLM-regulated gene expression and the full scope of MDLM-regulated genes remain unknown.

In addition to the CO-FT, PIF regulatory modules, and MDLM, it has been recognized that the circadian clock and circadian clock-controlled genes exhibit phase delays as photoperiod lengthens [23]. Models predict that the multiple interlocking feedback loops of the clock allow for clock genes to track dusk as it delays, relative to dawn [24]. Recently, *EMPFINDLICHER IM DUNKELROTEN LICHT 1* (*EID1*) was shown to be required for photoperiodic response of the circadian clock in tomato, but detailed mechanistic understanding of this phenomenon is lacking in many plants [25].

In the last 30 years, transcriptomics has emerged as an important tool for understanding the breadth of photoperiodic gene regulation. Subtractive hybridization was first used to identify photoperiod-regulated genes involved in flowering time [11], and subsequently microarray was used to identify local and global gene expression changes in response to the floral transition [26,27]. Additionally, microarrays were used to track gene expression changes in *Arabidopsis* at dusk and dawn under many photoperiods, and time course studies provided a view of the genes that exhibited altered phasing under long- and short-day photoperiods [23,28]. Transcriptomics have now been implemented to study photoperiodic gene expression in *Arabidopsis hallerrii* [29], *Panicum hallii* [30], wheat [31,32], *Medicago* [33], sugarcane [34], and soybean [35]. These studies have revealed that photoperiodic gene expression changes mainly manifest as changes in phase (i.e., clock genes) or amplitude (i.e., *FT* or *PP2-A13*).

Recently, 2 studies reanalyzed older transcriptomic data and uncovered new photoperiod measurement mechanisms. A meta-analysis of *Arabidopsis* transcriptomics led to the discovery that phytochrome A is important for light sensing in short days [36]. Additionally, a study using relative daily expression integral (rDEI = sum of 24 h of expression in condition 1/sum of 24 h of expression in condition 2) followed by expression pattern clustering identified short day-induced genes in *Arabidopsis* and precipitated the discovery of the MDLM system [21].

Despite these inroads towards understanding photoperiod-responsive transcriptional systems, we still have an incomplete understanding of the genes and cellular processes regulated by photoperiod and the scope of potential photoperiod measuring systems in plants. Deficiencies in studying photoperiodic transcriptomes have been caused by variation in sampling frequency, time points, growth conditions, photoperiod length, and ease of data access. To address this, we performed RNA-sequencing on a 24-h *Arabidopsis* time course encompassing 3 photoperiods: 8 h light followed by 16 h dark (8L:16D), 12L:12D, and 16L:8D. We used an rDEI and pattern clustering pipeline to identify and classify photoperiod-regulated genes. Furthermore, *cis*-element analysis was performed to provide further evidence that co-clustered genes share known and de novo transcription factor-binding elements that point towards distinct photoperiodic transcriptional systems. Additionally, Gene Ontology (GO) and Kyoto Encyclopedia of Genes and Genomes (KEGG) enrichment analyses identified a host of cellular pathways that are potentially controlled by photoperiod in *Arabidopsis*. We then followed one important cellular pathway, phenylpropanoid biosynthesis, and found a complex regulatory network that differentially controls separate branches of this pathway. Finally, we present “Photo-graph,” an app for user-friendly visualization of photoperiod data. Together, this work provides a comprehensive examination of photoperiod-responsive transcriptional systems in *Arabidopsis* and suggests that a multitude of networks control important cellular pathways in response to daylength.

## Results

### A time course transcriptome dataset for identifying photoperiodic genes

**Fig 1:**
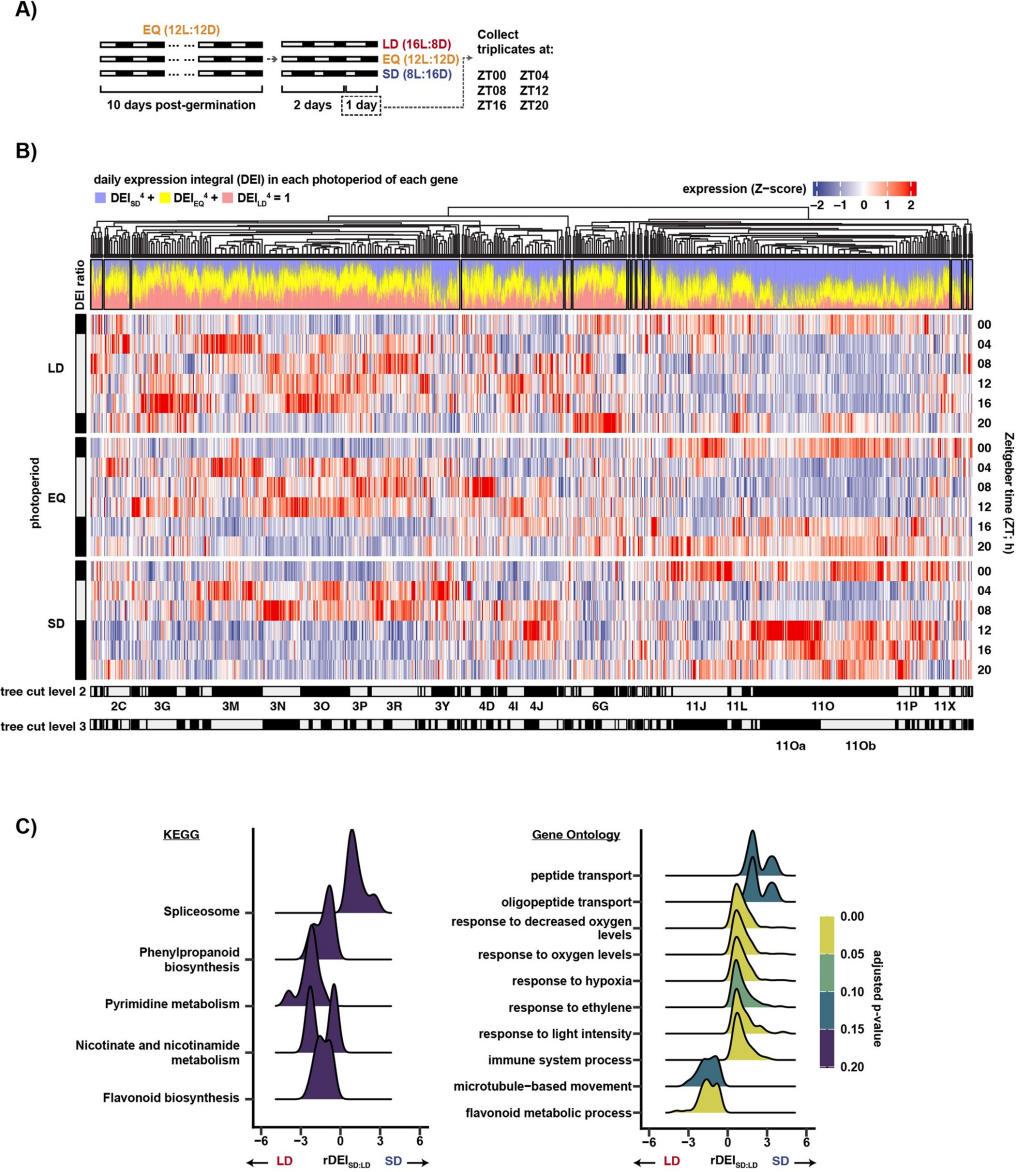
Comparison of gene expression between 3 photoperiods. (**A**) The experimental design. Gray and dark bars represent light and dark periods, respectively. The first time point is zeitgeber time hour 0 (ZT00). In this experiment, zeitgeber time is equal to the number of hours from dawn. (**B**) (Top) Agglomerative clustering of 8,293 photoperiodic genes. (DEI ratio) Stacked bar chart of the DEI of each gene, transformed with: (DEI_SD_)^4^/k + (DEI_EQ_)^4^/k + (DEI_LD_)^4^/k = 1. (Middle) Heatmap of scaled gene expression pattern. (Bottom) Assignment of subgroups with dynamic tree cut, with depth = 2 or 3 (**S5 Data**). Position of subgroups mentioned in text are labeled. (**C**) Ridgeplot showing the distribution of the leading edge genes of top GO and KEGG pathway terms of GSEA using DEI ratio (rDEI) between LD and SD as ranking metric (**S6 Data**); *p*-value was adjusted using the Benjamini–Hochberg procedure. Only the top 10 terms ordered by absolute normalized enrichment score (NES) that pass the threshold of adjusted *p*-value < 0.2 are shown. DEI, daily expression integral; GO, gene ontology; GSEA, gene set enrichment analysis; KEGG, Kyoto Encyclopedia of Genes and Genomes; LD, long day; NES, normalized enrichment score; rDEI, relative daily expression integral; SD, short day.

With the goal of identifying photoperiod-responsive transcriptional systems in *Arabidopsis*, we wanted to isolate direct target genes of these systems while avoiding downstream developmental, age-related, and tissue type differences that could arise from constant growth in different photoperiods. We designed a growth regime to capture direct target genes: *Arabidopsis* seedlings were grown for 10 days in equinox (EQ; 12L:12D) to ensure equivalent growth, and then shifted to short day (SD; 8L:16D), EQ, or long day (LD; 16L:8D) for 2 days prior to collection of whole seedlings, including shoot, hypocotyl, and root (**Fig 1A**). We wanted to ensure that the shifted photoperiod growth regime was capturing known photoperiodic gene expression changes. Thus, we also collected samples from plants grown in constant SD and LD to compare gene expression between the shifted and constant photoperiod growth regimes (**S1 Fig**). Triplicate samples were harvested at 4-h intervals for both experiments. We chose 2 well-studied photoperiod-regulated genes: the CO-regulated gene *FT* and the MDLM-regulated gene *PP2-A13*, and qRT-PCR confirms that they are expressed similarly between plants grown in the shifted photoperiod versus constant photoperiod conditions (**S2 Fig and S1 Data**). This validates the ability of our approach to identify direct target genes of known photoperiod-responsive transcriptional systems in the absence of developmental changes. Thus, we performed RNA-sequencing on samples from plants grown in the shifted photoperiods (**S2 and S3 Data**).

We used a multitiered filtering approach to identify biologically relevant transcriptional systems from this dataset. This included an initial inclusive identification of photoperiod-regulated genes, clustering of co-expression groups, calculation of daily expression integral, false-positive and false-negative assessment using qRT-PCR, and enrichment analysis of functional annotation and promoter *cis*-elements. We first identified a set of 8,293 genes that are differentially expressed (*p* < 0.2) in at least 1 time point between any 2 photoperiods (**S3A, S3B, and S4 Data; Methods**). We next clustered the genes based on their daily expression patterns using affinity propagation and subsequently merged them with exemplar-based agglomerative clustering [37]. This method assembled the photoperiod-regulated genes into 14 clusters (C1-C14) (**Figs 1B and S4 and S5 Data)**. In addition to clustering, we calculated the daily expression integral (DEI) ratio between the 3 photoperiods by summing expression for each transcript across each photoperiod time course and then calculating the scaled percent expression in each photoperiod (**Fig 1B** “DEI ratio”). This provides a simple metric and visual method to determine the photoperiod in which the transcript is most highly expressed: blue for SD, yellow for EQ, and red for LD.

Our goal for the initial filtering was to enrich for genes with varying expression across photoperiods, thus the use of *p*-value < 0.2 (8,293 differentially expressed genes) followed by stringent downstream analyses. To test for false positives and false negatives, we filtered our data with a more stringent threshold of FDR < 0.05 (2,668 differentially expressed genes; **S3C and S3D Fig**), and then used qRT-PCR to test the expression of genes that are differentially expressed at a threshold of FDR < 0.05 (*4CL*, *CHS*, *KMD1*, *PAL1*, *PP2-A13*, *RPL10*) and those that are differentially expressed only at *p* < 0.2 (*ALAAT1*, *COR27/28*, *DREB1A*/*1B*, *EXL1*, *LHCA1/3*, *RPL5A*) (**S2 Fig**). We find the genes that are differentially expressed only at *p* < 0.2 in the RNA-sequencing analysis show photoperiodic regulation in qRT-PCR, suggesting a high false-negative rate at FDR < 0.05. This validates our decision to start with an inclusive cutoff prior to clustering and functional analyses, although we have included gene numbers at the more restrictive cutoff (FDR < 0.05) for reference in subsequent discussion of important gene groups (**Table 1** and **S4 Data**).

**Table 1:** Description of the 19 gene subgroups with at least 100 genes.

Gene group	Number of genes (*p* < 0.2)	Number of genes (FDR < 0.05)	Mean log_2_(rDEI_SD:LD_)	Mean log_2_(rDEI_SD:EQ_)	Mean log_2_(rDEI_EQ:LD_)	Notable genes
2C	207	36	−0.348	−0.167	−0.180	-
3G	277	119	−0.675	−0.291	−0.384	-
3I	133	28	−0.268	−0.207	−0.061	AT1G14320 | *RPL10* AT1G72370 | *RP40* AT2G39460 | *RPL23A* AT3G25520 | *RPL5A*
3M	492	156	−0.235	−0.138	−0.097	AT1G22640 | *MYB3* AT1G29920 | *CAB2* AT1G32640 | *MYC2* AT1G51680 | *4CL1* AT2G37040 | *PAL1* AT3G51240 | *TT6* AT4G39800 | *MIPS1* AT5G13930 | *CHS* AT5G64040 | *PSAN*
3N	358	153	−0.166	−0.076	−0.09	AT2G40080 | *ELF4* AT4G25480 | *DREB1A* AT4G25490 | *DREB1B*
3O	482	247	−0.404	−0.25	−0.154	AT1G12140 | *FMO* AT1G24100 | *UGT74B1* AT1G74090 | *SOT18* AT2G04030 | *HSP90*.*5* AT4G24190 | *HSP90*.*7*
3P	166	72	−0.621	−0.481	−0.14	-
3R	439	162	−0.129	−0.01	−0.119	AT1G29910 | *CAB3* AT1G29930 | *CAB1* AT1G61520 | *LHCA3* AT2G43010 | *PIF4* AT3G61470 | *LHCA2* AT3G47470 | *LHCA4* AT3G54890 | *LHCA1* AT5G62430 | *CDF1*
3Y	227	39	0.469	0.447	0.022	AT1G17290 | *ALAAT1* AT4G08870 | *ARGAH2* AT4G39950 | *CYP79B2*
4D	126	10	−0.29	−0.491	0.201	-
4I	161	38	−0.291	−0.165	0.126	AT5G60100 | *PRR3*
4J	316	115	0.235	0.248	0.013	AT2G25930 | *ELF3* AT3G26740 | *CCL* AT4G33980 | *COR28* AT5G42900 | *COR27*
6F	117	19	−0.522	−0.228	0.294	-
6G	202	53	−0.522	−0.376	0.147	-
11J	521	111	0.254	0.068	0.186	AT1G01060 | *LHY* AT2G46830 | *CCA1* AT5G02840 | *RVE4* AT5G17300 | *RVE1*
11L	100	24	−0.121	−0.063	0.058	-
11O	1,398	887	0.557	0.327	0.23	AT1G25560 | *TEM1* AT1G80440 | *KMD1* AT3G47340 | *DIN6* AT3G61060 | *PP2-A13* AT5G54080 | *HGO*
11Oa	587	395	0.76267877	0.48590304	0.2767757	AT1G80440 | *KMD1* AT3G47340 | *DIN6* AT3G61060 | *PP2-A13* AT5G54080 | *HGO*
11Ob	711	434	0.36874602	0.1847722	0.1839738	AT1G25560 | *TEM1*
11P	111	37	0.687	0.394	0.293	-
11X	107	12	−0.15	−0.179	0.03	-

EQ, equinox; LD, long day; rDEI, relative daily expression integral; SD, short day.

We next performed gene set enrichment analysis (GSEA) by ranking the photoperiod-regulated genes by their DEI and then tested GO and KEGG terms for association with the ranking [38]. This allows us to visualize cellular pathways that are enriched in SD, EQ, and LD (**Figs 1C and S5 and S6 Data**). Top annotation terms associated with SD-induction are “response to hypoxia,” “valine, leucine, and isoleucine degradation,” “spliceosome,” and “peptide transport,” while those with LD-induction fall into 3 biological categories: phenylpropanoid biosynthesis, NAD biosynthesis, and microtubule-based movement. “Pentose and glucuronate interconversions” is associated with EQ-induction. Some of these categories were similarly enriched in previous studies, providing confidence that our results are biologically relevant [21,39].

Some of the annotation terms identified, e.g., “response to hypoxia” and “phenylpropanoid biosynthesis,” could be associated with stress responses. To test whether a general stress response was triggered by the shifted photoperiod growth regime, we chose marker genes of various biological processes and compared expression with that of the plants grown in constant photoperiod. The constant photoperiod growth regime has no shift in dusk timing that could cause a stress response. We chose genes that respond to hypoxia, cold, dehydration, and light, and genes involved in phenylpropanoid biosynthesis and ribosomal processes. We observe a striking resemblance of expression patterns with the exception of slightly higher SD expression level in genes related to light response (*LHCA1*/*3*) and phenylpropanoid biosynthesis (*4CL1*, *CHS*, and *PAL1*) in the constant photoperiod regime (**S2 Data**). This indicates that there was not a general stress response caused by the photoperiod shift.

We next assessed the clusters based on expression pattern. Two large clusters, C3 (*n* = 3,157) and C11 (*n* = 2,883), encompass 73% of the photoperiod-regulated genes. C3 contains genes highly expressed in the light, which generally results in higher expression in LD as measured by DEI (**Fig 1B**, “DEI ratio”). C11 contains genes highly expressed in the dark, which in general results in higher expression in SD as measured by DEI (**Fig 1B**, “DEI ratio”). This light-dark division is apparent in the principal component analysis, which oriented samples by the light condition and the time of day (**S6 Fig**). Other prominent clusters include C4 (*n* = 982), which shows high expression in the mid-day, i.e., zeitgeber time 08 hour (ZT08) and ZT12, and C6 (*n* = 519), which has a prominent peak at ZT20 in LD (**Fig 1B**).

**Fig 2:**
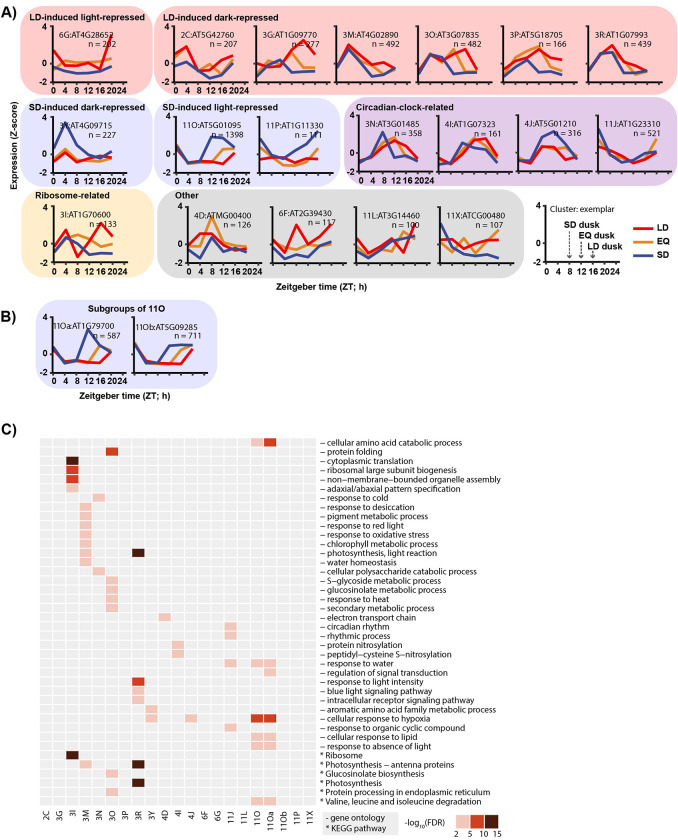
Photoperiod-regulated genes display expression patterns and associate with biological processes. (**A**) Gene exemplars of major subgroups (at least 100 genes) generated by affinity propagation (**S5 Data**); *n* refers to the number of genes in subgroup. Blue: SD expression; orange: EQ expression; red: LD expression. (**B**) Gene exemplars of divisions of 11O, 11Oa, and 11Ob, selected by increasing the depth of dynamic tree cut from 2 to 3 (**S5 Data**). (**C**) Significant enrichment of GO and KEGG pathway terms in gene subgroups (false discovery rate < 0.05) (**S6 Data**); *p*-value was adjusted using the Benjamini–Hochberg procedure. GO and KEGG term enrichment of divisions of 11O, 11Oa, and 11Ob were also shown. EQ, equinox; GO, Gene Ontology; KEGG, Kyoto Encyclopedia of Genes and Genomes; LD, long day; SD, short day.

We noted that a diverse group of daily expression patterns were housed together within the larger clusters, including C3 and C11. These could represent genes expressed under the control of distinct photoperiod transcriptional systems. To extract subgroups within the 14 large clusters, we used dynamic tree cut [40] and affinity propagation to select gene exemplars that best describe each subgroup (**Figs 1B bottom, 2A, S2, S7, S8 and Table 1**). This separated all photoperiod-regulated genes into 99 subgroups with a mean size of 84 genes (**S5 Data**). We identified 19 major subgroups containing at least 100 genes at *p* < 0.2, all of which are still present at FDR < 0.05. Although smaller gene groups may be biologically meaningful, we focus downstream analyses on larger groups that might represent major photoperiod gene expression systems in *Arabidopsis*. Importantly, tuning the dynamic tree cut at various depths breaks down the largest subgroup 11O (*n* = 1,398) into 2 large and visually distinctive groups, which we termed 11Oa (*n* = 587) and 11Ob (*n* = 711), and other small subgroups (**Fig 2B**). While both groups are dark induced and light repressed, 11Oa has a strong post-dusk induction peak, similar to genes controlled by MDLM, while 11Ob has a weaker post-dusk induction and a dominant dawn-phased peak, resulting in even expression across the night.

To assess the validity of our dataset, we examined enrichment of published CO-regulated genes in our subgroups under the presumption that CO-regulated genes would be enriched in the LD-induced clusters [12]. As predicted, CO-regulated genes are grouped in the LD-induced groups 3M and 3O, giving us confidence that our dataset can detect transcriptional networks from known photoperiod measurement systems (FDR < 0.05, hypergeometric test) (**S7 Data**). We also compared our data to genes that are differentially regulated in the *pifq* mutant [41], and as expected group 3R is enriched in genes up-regulated in the *pifq* mutant, in agreement with the enrichment of light-induced genes in that cluster (**Table 1 and S7 Data**). The MDLM-regulated genes are also located in the appropriate subgroups. *PP2-A13* is located in 11Oa [21] and *MIPS1* is located in 3M [22], which match the previously demonstrated gene expression patterns (**Table 1**).

**Fig 3:**
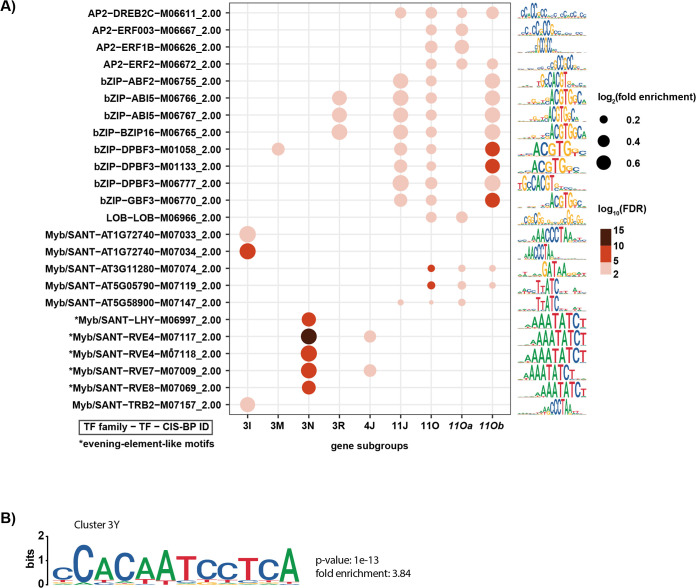
Enrichment of AP2/ERF-, bZIP- and Myb/SANT-class TFBSs in photoperiod-regulated genes. (**A**) Significant enrichment of TFBSs in CIS-BP in promoters of gene subgroups, including 11Oa and 11Ob (**S8 Data**). Only the top 3 enriched motifs of each subgroup that pass the statistical threshold (false discovery rate < 0.001) are shown; *p*-value was adjusted using the Benjamini–Hochberg procedure. Dot size represents fold enrichment and color represents statistical significance of enrichment. Sequence logos of the corresponding motifs are shown on the right. Sequence logos are scaled to the information content of motif bases. (**B**) Top de novo motifs of cluster 3Y (**S8 Data**). The unadjusted *p*-values and fold enrichment reported by HOMER are shown. Sequence logos are scaled to the information content of motif bases. TFBS, transcription factor binding site.

We performed enrichment tests of GO terms and KEGG pathways on the 19 clusters with at least 100 genes (**Fig 2C and S6 Data**). This allows us to identify potential cellular pathways regulated by photoperiod and to characterize clusters based on cellular function. Additionally, we performed motif enrichment analysis on the gene promoters from each subgroup using transcription factor binding sites (TFBSs) in the CIS-BP database (**Fig 3A and S8 Data**) [42], in order to further characterize each subgroup based on enrichment of common regulatory motifs and assess their biological relevance.

In the following sections, we will describe the large subgroups and provide evidence for their classification into separate photoperiodic transcriptional groups.

### Circadian clock genes

Lengthening photoperiod causes delayed phase of circadian clock genes [23]. Four subgroups have evidence that prompted us to classify them as clock genes associated with photoperiod: 3N, 4I, 4J, and 11J (**Fig 2A**). 3N, 4I, and 4J have a single expression peak phased to midday, while 11J has a single expression peak phased to dawn. Phase analysis shows that groups 3N and 4I show the hallmark phase delay associated with clock genes responding to lengthening photoperiod (**S9 Fig and S9 Data**). Groups 4J and 11J do not show the same change in phase but show an increase in magnitude in SD, resulting in a slight increase in the ratio of SD DEI to LD DEI (rDEI_SD:LD_) (**Table 1**). All 4 clusters contain known clock genes. 4J and 11J are enriched in GO terms “circadian rhythm” and “rhythmic processes” (**Fig 2C**). 3N is enriched for the GO terms “response to cold” and “cellular polysaccharide catabolic process”. 4I is enriched for GO terms related to protein nitrosylation. 3N and 4J show statistically significant enrichment of the evening element, a well-studied clock *cis*-element [43] (**Fig 3A**). 11J shows enrichment of the bZIP binding core sequence, ACGT [44]. Our results identified 4 photoperiodic subgroups that are likely linked to the circadian clock. Two showed the hallmark change in phase associated with the clock response to photoperiod and 2 showed no change in phase but slight amplitude increases in response to photoperiod. Together, the identification of photoperiod-regulated clock genes and the clock *cis*-elements confirms that our dataset can identify known photoperiod responsive transcriptional networks.

### Short day-induced genes

In the clustering performed here, 11O is the largest of the SD-induced subgroups, as determined by rDEI (**Fig 1B** and **Table 1**). However, further dynamic tree cutting suggests that 11O contains 2 separate expression groups, which we termed 11Oa and 11Ob (**Fig 2B**). Both groups have biphasic expression in SD and are repressed in the light. 11Oa is distinguished by a dominant post-dusk peak and a weaker dawn-phased peak, while 11Ob is characterized by a weaker post-dusk peak and a more prominent dawn-phased peak.

The 11Oa subgroup contains the MDLM-regulated gene *PP2-A13*, and the expression pattern of this subgroup is identical to the *PP2-A13* daily expression pattern shown previously [21]. Furthermore, it contains genes shown to be important for short-day physiology (*PP2-A13*, *EXORDIUM-LIKE 1*, and *HOMOGENTISATE 1*,*2-DIOXYGENASE*) [21,45]. In support of its role in short-day plant physiology, 11Oa is enriched with genes involved in hypoxia, response to absence of light and amino acid catabolism (**Fig 2C**). The enrichment of hypoxia responsive and amino acid metabolism genes suggests a response to lower energy availability in SD. Breakdown of branched chain amino acids is an energy scavenging mechanism and is a major response to both hypoxia and extended darkness when energy is limited [46–48]. Conversely, 11Ob has a weaker post-dusk expression peak, but a more dominant dawn-phased expression peak (**Fig 2B**). 11Ob contains *TEMPRANILLO1* (*TEM1*), a gene known to repress *FT* expression in short days, but 11Ob shows no enrichment of any individual cellular pathways (**Table 1 and Fig 2C**) [49,50].

We next inquired whether the 2 subgroups have enrichment of shared or distinct *cis*-elements. The entire 11O subgroup has 2 enriched *cis*-elements: the bZIP TFBS resembling the G-box (core sequence CACGTG) [44] and the AP2/ERF TFBS resembling the GCC-box (core sequence AGCCGCC) [51] (**Fig 3A**). Interestingly, 11Oa has the dominant post-dusk expression peak but lacks enrichment of the bZIP sites, only containing that of the AP2/ERF sites. 11J has genes that are dawn-phased and is enriched with the bZIP sites but not the AP2/ERF binding sites. 11Ob contains genes that have the post-dusk peak and the dawn-phased peak and is enriched with both AP2/ERF and bZIP sites. This correlation may indicate that the AP2/ERF sites are important for post-dusk phasing in short days, and the bZIP sites are important for dawn phasing.

Cluster 3, which contains subgroups mostly induced in LD, also contains the outlier subgroup 3Y that is induced in SD (**Fig 2A**). This subgroup demonstrates monophasic peaking at ZT4 that increases in amplitude in short days. This SD-induction in the light rather than the dark makes 3Y unique. It was also enriched in genes involved in hypoxia (**Fig 2C**). We were unable to identify any know *cis*-regulatory elements that were enriched in 3Y (**Fig 3A**). A search for de novo motifs identified 1 strongly enriched element containing the sequence CCACAATCCTCA (**Fig 3B**).

These results suggest that there are potentially 3 transcriptional systems controlling 3 major SD-induced gene expression programs. One is characterized by strong post-dusk induction and is enriched with an AP2/ERF binding site. A second potential program is exemplified by the dawn-phased genes enriched with the bZIP core. bZIP transcription factors (TFs) play a number of roles in plants, including control of the circadian clock and light signaling [52,53]. A third subgroup, 3Y, shows high amplitude SD expression at ZT4 and contains a de novo motif. Little is known about this smaller transcriptional system, but the enrichment of important cellular pathways, such as hypoxia and amino acid metabolism, suggests this may be important for plants grown in SD.

### Long day-induced genes

The majority of LD-induced genes reside in cluster 3, but in contrast to the SD-induced genes, cluster 3 contains a greater number of smaller subgroups rather than 1 large subgroup like 11O (**Fig 2A**). This could indicate that multiple photoperiod-measuring systems control gene expression in long days. This is supported by evidence showing that the MDLM and CO systems can cause similar photoperiodic gene expression changes (**S7 Data**) [22]. To determine if there are possible transcriptional systems that are driving LD-induced gene expression, we further analyzed 5 major subgroups from cluster 3 (3G, 3M, 3O, 3P, and 3R). All are expressed mainly in the light period of the day, hence their presence in cluster 3, but only 3M, 3O, and 3R are strongly repressed by the dark in all 3 photoperiods (**Fig 2A**). 3M is enriched in genes related to pigment metabolic process, desiccation, chlorophyll metabolic process, response to oxidative stress, response to red light, and water homeostasis (**Fig 2C**). 3O is enriched in genes involved in protein folding, glucosinolate metabolic process, response to heat, and protein processing in the endoplasmic reticulum. 3R is enriched in genes involved in blue light signaling, response to light intensity, and photosynthesis; *cis*-element analyses did not identify any single site enriched in subgroups 3G, 3O, and 3P (**Fig 3A**). Conversely, 3M and 3R are weakly enriched in bZIP sites. 3M and 3R have a similar expression pattern, resembling that of the MDLM-controlled gene *MIPS1*, which is located in 3M [22]. Because of the shared enrichment of *cis*-elements in the subgroups that contain the LD and SD MDLM genes, it is possible that the same families of TFs are in play to control gene expression in both photoperiods.

In addition to the aforementioned subgroups that result in higher gene expression in LD and are expressed mostly in the light period, there is 1 night-phased LD-induced subgroup, 6G (**Fig 2A**). Also displaying higher expression in LD is the day-phased subgroup, 2C, which achieves this through a peak magnitude increase at ZT4. Similar to 3G, 6G and 2C have no enrichment of any biological pathways or known *cis*-elements (**Figs 2C, 3A and 3B**).

In sum, we can identify target genes from known photoperiod measurement systems intermingling in the large C3 subgroup. The CO-regulated genes are spread across many subgroups, but the MDLM-regulated genes are clustered in 3M and 3R, based on *cis*-element enrichment analysis and expression pattern. Additionally, there may be photoperiod measurement systems that have not been identified that could account for other modes of expression.

### Photoperiod regulation of ribosomal genes

One subgroup, 3I, is defined by a ZT8-specific trough in LD that causes a biphasic expression pattern only in LDs (**Fig 2A**). Furthermore, this subgroup is strongly enriched with genes involved in ribosome biogenesis and translation (**Fig 2C**). In support of this, *cis*-element analysis showed enrichment of the binding site for the Myb-type TF TELOMERE REPEAT BINDING FACTOR (TRB) 2 and AT1G72740 (**Fig 3A**), both belonging to a TF family of evolutionarily conserved regulators of ribosome gene expression [54,55]. This subgroup is unique because it was the only major subgroup defined by an expression trough rather than an expression peak (**S8 Fig**). It will be worthwhile in the future to determine if the TRB site plays a role in this process.

### Equinox induced-genes

It is conceivable, and demonstrated in some cases, that some biological processes may be induced or repressed specifically in the equinox photoperiods in plants [3]. We included a 12L:12D equinox photoperiod in order to test this idea. We found few genes that were expressed highly in LD and SD but repressed in EQ, but we found a greater number of genes that are expressed specifically in EQ but reduced in LD and SD. These included clusters 3A (*n* = 82), 4C (*n* = 92), 4D (*n* = 126), 9A (*n* = 56), 11B (*n* = 41), and 11C (*n* = 39). These were spread across a variety of peak times, but only 4D contained more than 100 genes (**S7 and S8 Figs**). In 4D, we found enrichment of electron transport chain genes, suggesting it is important for photosynthetic processes (**Fig 2C**). We did not identify additional elements that point towards an EQ-specific mechanism, but this could be investigated further in follow-up studies.

In the previous sections, we defined 19 major photoperiod expression patterns and tentatively linked 13 to biological processes or *cis*-elements. Notably, the 6 other patterns did not show enrichment of annotation or promoter *cis-*elements, and further evidence is required to suggest them as distinct photoperiodic transcriptional systems. What is clear is that photoperiod gene expression changes can manifest with a diverse array of daily expression patterns that cannot be accounted for with our current knowledge of photoperiod measurement systems in plants.

### Photoperiodic control of phenylpropanoid biosynthesis

**Fig 4:**
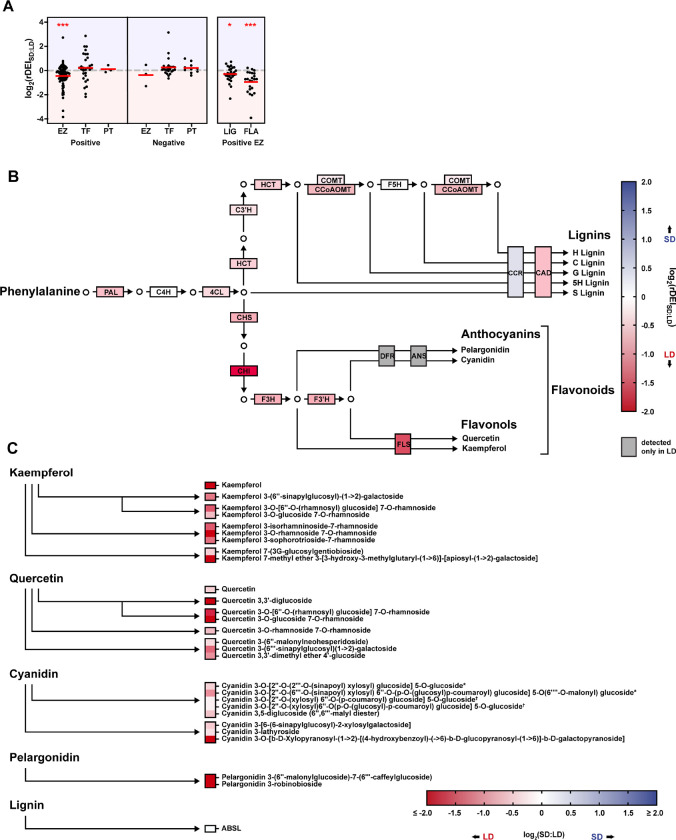
Photoperiod regulates phenylpropanoid gene expression and metabolite accumulation. (**A**) Distribution of rDEI_SD:LD_ in genes involved in phenylpropanoid production (*n* = 189) (**S10 Data**). Genes are grouped according to positive/negative effect on the phenylpropanoid pathway, molecular function as an EZ, TF, or PT regulator, or LIG vs. FLA branch. Red bars indicate mean. *, *p* ≤ 0.05, ***, *p* ≤ 0.0001 (1 sample Wilcoxon signed rank test). Blue shading, SD-induced genes, or compound accumulation; red shading, LD-induced genes or compound accumulation. (**B**) Simplified phenylpropanoid biosynthesis pathway (**S10 Data**). Box labeling corresponds to biosynthetic enzyme names; box shading corresponds to log_2_(rDEI_SD:LD_) of the coding biosynthetic gene. (**C**) Precursor modifications and relative compound accumulation (**S11 Data**). Box labeling corresponds to compound name; box shading corresponds to SD:LD relative peak area ratios. *,†The indicated pairs of compounds could not be fully resolved from one another. EZ, enzyme; FLA, flavonoid; LD, long day; LIG, lignin; PT, post-translational; rDEI, relative daily expression integral; SD, short day; TF, transcription factor.

We next tested if our pipeline is effective at identifying and classifying bona fide photoperiod-regulated cellular pathways. GSEA identified phenylpropanoid biosynthesis as one of the top cellular processes enriched with photoperiod-regulated genes (**Fig 1C**). Anthocyanin production is controlled by photoperiod in many plants [56], but in *Arabidopsis* it is not clear if they are induced by short or long days, nor if other byproducts of the phenylpropanoid pathway, such as other flavonoids or lignin, are also regulated by photoperiod [36,57]. To address this, we curated a catalog of genes involved in phenylpropanoid synthesis in *Arabidopsis* using KEGG, GO, and an extensive literature search (**S10 Data**). Each gene was annotated according to its predicted effect on the phenylpropanoid pathway, mode of action, and the branch of the pathway in which it acts. To determine how photoperiod regulates the transcription of positive and negative regulators of the phenylpropanoid pathway, both groups were plotted according to their rDEI_SD:LD_ (**Fig 4A**). The expression of positive regulators of phenylpropanoid biosynthesis, especially that of the flavonoid branches, was found to be significantly higher in LD. To visualize the seasonal induction of phenylpropanoid genes more precisely, we mapped the rDEI_SD:LD_ of key enzymes to the phenylpropanoid biosynthesis pathway (**Figs 4B and S10**). Notably, enzymes specific to the flavonoid branches are more highly LD-induced than those specific to the lignin branch, which also contains the SD-induced gene *CINNAMOYL COA REDUCTASE 1* (*CCR1*) (**S11 Fig**).

Our expression analyses indicate that flavonoids are potentially induced in LDs, while the photoperiodic control of the lignin branch is weaker. To test if the observed pattern of phenylpropanoid gene expression corresponds to seasonal regulation of metabolites, we quantified various phenylpropanoid compounds in LD- and SD-grown plants (**Fig 4C and S11 Data**). In agreement with observed gene expression patterns, liquid chromatography–mass spectrometry (LC-MS) detection revealed higher levels of 18 flavonoid compounds in LD rather than in SD photoperiod (FDR < 0.05, Student’s *t* test). Again, in agreement with gene expression, quantification of acetyl bromide soluble lignin (ABSL) found lignin polymer accumulation to be unaffected by photoperiod (*p* > 0.1, Student’s *t* test) (**Fig 4C and S11 Data**). Together, these data provide a holistic view of the photoperiodic regulation of phenylpropanoids and suggest differential regulation of the lignin and anthocyanin/flavonol branches of the phenylpropanoid pathway with respect to photoperiod. Specifically, anthocyanins and flavonol genes are induced in LDs and the corresponding metabolites respond accordingly, while the lignin genes do not show consistent photoperiodic regulation and lignin content in cells remains constant across photoperiods.

### The “Photo-graph” app provides a user-friendly way to access and analyze photoperiod transcriptomics data

**Fig 5:**
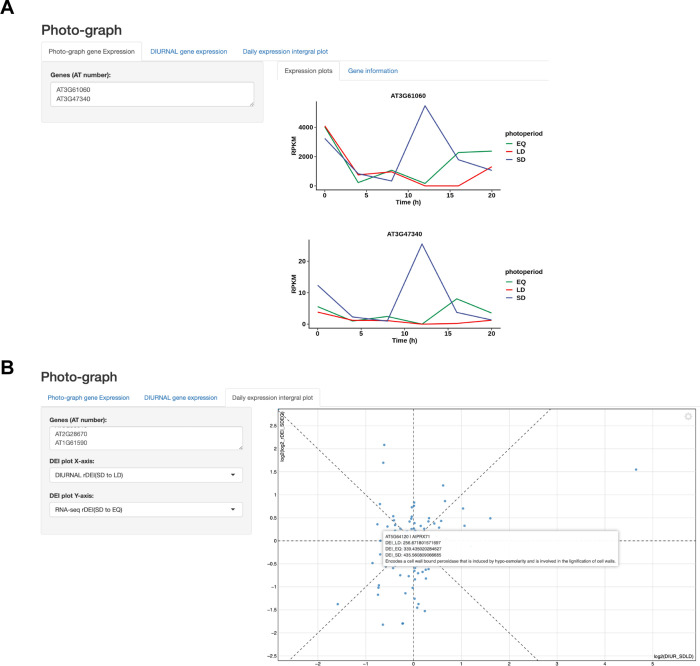
The “Photo-graph” app provides a user-friendly visualization of gene expression patterns. (**A**) Visualization of RNA-sequencing expression pattern. (**B**) Plot of rDEI_SD:LD_ in this dataset against the rDEI_s_. rDEI, relative daily expression integral.

The daily expression pattern and rDEI are informative for understanding photoperiodic gene expression, but there is currently not a user-friendly online tool to visualize this. We created an app and named it “Photo-graph” (https://gendron-lab.shinyapps.io/PhotoGraph/) that allows access to the data with a user-friendly interface. Users may query the gene expression pattern and rDEI of any detectable *Arabidopsis* genes through simple input of TAIR identifiers (**Fig 5A**). Additionally, data can be plotted by rDEI, allowing for easy identification of genes induced in specific photoperiods (**Fig 5B**).

Furthermore, the Photo-graph app has the potential to display any photoperiod-specific time course data from multiple sources and is not restricted by organism or data type. We show this by including long- and short-day microarray data from the DIURNAL site [23]. One can choose to look at expression of their gene of interest in previously published microarray data alongside the RNA-sequencing data provided here.

## Discussion

Cellular and physiological health in plants relies on accurately measuring daylength to predict seasonal change. In plants, photoperiod measurement is particularly important not only for ensuring fecundity in offspring, but also for optimizing fitness and growth. Studies of flowering time in plants have dominated research in photoperiodism, but here we provide transcriptomic data and analyses that indicate that multiple transcriptional systems are communicating photoperiod information to control a wide variety of important cellular processes through regulation of gene expression.

Using an agglomerative approach, we identified that thousands of *Arabidopsis* genes have expression changes dependent on photoperiod. Photoperiodic gene expression changes can be conceptually grouped into 2 broad categories: changes in phase and changes in amplitude, demonstrating the need to analyze time course data that spans at least 24 h. Next, using a dynamic tree cutting approach, we were able to group the genes into 19 co-expressed subgroups that encompass diverse expression patterns (**Table 1** and **Fig 2A**).

Perhaps most strikingly, many photoperiod-regulated genes fall into 2 large classes: genes induced in light and repressed in dark, and the opposite, genes induced in dark and repressed in light. Interestingly, within these categories, there seem to be multiple transcriptional systems at play. For example, genes induced in SD in the dark fall into 3 major categories: genes containing a dominant post-dusk peak of expression, genes containing a dominant dawn-phased peak of expression, and genes with both. This aligns with *cis*-element enrichment, suggesting that bZIP binding sites are enriched in dawn-phased genes and AP2/ERF binding sites are enriched in post-dusk phased genes (**Fig 3A**). It is tempting to speculate that these enriched binding sites are indicating the transcriptional control points for genes that are regulated by MDLM, given that genes such as *PP2-A13* fall into these categories and are known MDLM targets [21] (**S12A Fig**).

Genes induced in LDs during daylight fall into a variety of subgroups. Intriguingly, subgroup 3M and 3R have very similar expression patterns and also show enrichment of the bZIP sites (**Fig 3A**). These clusters also contain genes known to be induced by MDLM in LDs, allowing us to speculate that MDLM may be utilizing the bZIP *cis*-elements for control of LD and SD genes (**S12B Fig**). It will be important in future studies to determine the TFs that bind them to provide insights into how MDLM controls gene expression in response to photoperiod. Outside of 3M and 3R, other LD light-induced subgroups showed apparent enrichment of genes that could benefit plant fitness in summertime (**Fig 2B**), but clearly enriched *cis*-elements were not apparent (**Fig 3A**). This may be due to the co-clustering of genes with similar expression patterns that are controlled by different photoperiod measuring systems. This is supported by evidence showing that CO-regulated genes are distributed across a variety of LD subgroups.

It is well known that circadian clock genes have delayed phases as days lengthen. In this study, we not only identified this class of genes, but also putative clock genes that display an amplitude increase in SDs and enrichment of the bZIP TFBS (**Figs 2A and S12C**). Together, the presence of these 2 classes indicate that the clock can respond to photoperiod through both phase and amplitude changes, suggesting that multiple mechanisms connect the clock to photoperiod. Future studies should focus on understanding the molecular components required for these changes.

Outside of these major expression groups there are also interesting smaller groups, such as SD-induced genes that are phased to the light period of the day or a cluster of genes defined by an LD trough that is enriched with ribosomal genes (**S12D Fig**). Similar to other photoperiod study systems, understanding these systems will require the development of tools where genetics and molecular biology can be used to study their photoperiodic expression in greater detail. But what is clear is that a variety of interesting and previously unrecognized photoperiod transcriptional systems are functioning in *Arabidopsis* and likely other plants as well.

In addition to LD and SD, we included an EQ time course in our studies to increase the resolution across different seasons. Although there were far fewer EQ-induced genes than LD- or SD-induced genes, EQ subgroups are enriched in genes involved in photosynthesis, matching the developmental strategy of an understory plant, such as *Arabidopsis*, which must often grow quickly in spring to beat shade produced by canopy trees (**S7 Fig**). Again, it will be interesting to create tools to track EQ-specific gene expression to understand how these patterns are controlled at a molecular level.

In addition to identifying a diversity of photoperiodic expression patterns, this work also enhances our knowledge of the cellular systems that are controlled by photoperiod. Importantly, we see a division of light-related and dark-related biological processes between the large clusters C3 and C11 (**Fig 2C**). Pathways related to photosynthesis, metabolism of pigments, and other secondary metabolites are enriched in the light-induced C3, whereas response to darkness and amino acid catabolic processes are enriched terms in C11.

Scrutiny into the subgroups shows that genes in some pathways are highly co-regulated. Genes that encode components of the photosynthetic machinery are enriched in 3M (e.g., *PSAN* and *CAB2*) and 3R (e.g., *LHCA1/2/3* and *CAB1/3*) (**Table 1**). The double peak subgroup 3M is also enriched in genes involved in oxidative stress, pigment metabolism, and desiccation. A major regulator of phenylpropanoid biosynthesis, *MYB DOMAIN PROTEIN 3* (*MYB3*) [58], and a key gene in the dehydration stress response, *MYC2*, can be found in 3M [59]. On the other hand, genes related to response to hypoxia, lipid and darkness are highly enriched in the double peak dark-induced subgroup 11Oa but not in 11Ob, which shows a similar pattern but without the SD-specific peak at ZT12. Importantly, this implies that the biological response towards the earlier dusk of SD is different from a general response to darkness.

Given our functional enrichment analysis identified a variety of potentially photoperiodic cellular processes, we sought to demonstrate the predictive power of the dataset. Much is known about the genes involved in phenylpropanoid biosynthesis and this pathway emerged as highly photoperiod regulated. Furthermore, reports have demonstrated photoperiodic regulation of anthocyanin, a major class of phenylpropanoids, but there are some discrepancies about whether they are induced in LDs or SDs [36,57]. Additionally, less is known about photoperiod regulation of 2 other major phenylpropanoid classes, flavonols and lignins. By creating a comprehensive catalog of phenylpropanoid genes and overlaying our photoperiod data, we were able to predict that anthocyanins and flavonols will be higher in LDs, while lignins will be less affected by photoperiod (**Fig 4B**). Quantitative measurements of these compounds confirmed this and demonstrated that our gene expression studies have the potential to predict physiologically relevant changes in response to photoperiod (**Fig 4C**).

In addition to generation of a dataset and analytical tools for photoperiod data, we also developed an app that can be used to visualize photoperiod expression data by plotting individual expression patterns or rDEI of gene groups. We named the app “Photo-graph.” This tool is not limited to *Arabidopsis* or plant time course data. We expect that other photoperiod time course data will be incorporated with this tool for use as a community resource as shown by our initial incorporation of photoperiod microarray data [23].

The presence of a diverse set of transcriptional systems and a large number of genes that respond to photoperiod indicate that plants are highly attuned to the length of day. Furthermore, this work provides a foundation on which to study the molecular components that drive this diverse set of seasonal expression patterns. This is especially important in the context of climate change where the photoperiod is rapidly becoming uncoupled from important seasonal signals, such as temperature and water availability. Understanding photoperiod-sensing systems will allow us to preempt the negative effect of climate change on plants.

## Materials and methods

### Plant materials and growth conditions

For RNA-sequencing, *Arabidopsis* Col-0 seeds were sterilized for 20 min in 70% ethanol and 0.01% Triton X-100 before being sown onto ½ Murashige and Skoog medium plates (2.15 g/L Murashige and Skoog medium (pH 5.6), Cassion Laboratories, cat. # MSP01, and 0.8% bacteriological agar, AmericanBio cat. # AB01185) lined with autoclaved filter papers. Seeds were stratified in dark at 4°C for 48 h before transferring to a growth chamber under 12L:12D photoperiod at 22°C and 130 μmol m^-2^ s^-1^ light intensity for germination. After germination, seedlings were kept in the same condition for 10 days. On day 11, the seedlings were transferred to 16L:8D, 12L:12D, or 8L:16D photoperiod. On day 13, whole seedlings with shoots and roots were harvested and snap frozen in liquid nitrogen. Approximately 50 seedlings from a single plate were pooled to generate 1 biological replicate, and 3 biological replicates in total were generated for each treatment group. For qRT-PCR, seedlings were stratified and germinated under identical conditions but were grown in 16L:8D or 8L:16D photoperiods for 13 days post-germination. For the LC-MS analysis and ABSL quantification, seedlings were stratified and germinated under identical conditions but were grown in 16L:8D or 8L:16D photoperiods for 14 days post-germination using the same growth medium.

### RNA extraction and library preparation

Total RNA was extracted from approximately 200 mg of pulverized whole *Arabidopsis* seedlings (shoot, hypocotyl, and root) using TRIzol reagent (Thermo Fisher, 15596026) according to manufacturer’s protocol. RNA samples were treated with RNase-free DNase (QIAGEN, 79254) to remove DNA contaminants. Protein contaminants were removed by extraction with phenol-chloroform mixture (phenol:chloroform:isoamyl-alchohol 25:24:1; Thermo Fisher, AM9730) followed by precipitation using 3 M sodium acetate solution. The resulting RNA was delivered to Yale Center for Genome Analysis for library preparation. Agilent Bioanalyzer was used to analyze sample quality. Samples with > 7.0 RNA integration number were used for the sequencing library preparation with the mRNA Seq Kit (Illumina, cat. # 1004814) following manufacturer’s instruction with alteration for mRNA extraction. mRNA was isolated from total RNA using 7 microliters of oligo dT on Sera-magnetic beads and 50 μl of binding buffer. The mRNA was fragmented in the presence of divalent cations at 94°C. Next, reverse transcription of the fragmented mRNA was performed with SuperScriptII reverse transcriptase (Thermo Fisher, cat. # 18064014), followed by end repair and ligation to Illumina adapters. The adaptor ligated DNA was amplified by PCR and then purified on Qiagen PCR purification kit (QIAGEN, 28104) to produce the libraries for sequencing. The libraries were sequenced on the Illumina NovaSeq6000 platform with S1 flow cells in paired end mode at 150 base pairs.

### RNA-sequencing analysis

Raw reads were trimmed using Trimmomatic (v.0.39) to remove low-quality reads and adapters [60]; the parameters were: -phred33 ILLUMINACLIP:TruSeq3-PE-2.fa:2:30:10:8 TRUE SLIDINGWINDOW:4:20 LEADING:5 TRAILING:5 MINLEN:36. The trimmed reads were aligned with HISAT2 (v.2.1.0) [61] with the parameters:—rna-strandness FR—no-mixed -I 100 -X 800 -x -p 10. The reads were aligned to a FASTA file containing the TAIR10 *Arabidopsis thaliana* genome (Ensembl version 47) and the ERCC spike-in sequence. Mapped reads were annotated with stringtie with the command: stringtie -v -e -B -G, using the TAIR 10 genome annotation. The resulting gene counts were formatted using the Stringtie function: prepDE.py.

### Identification of photoperiodic genes

Differential expression analysis was performed with the edgeR software [62]. Read counts of ERCC spike-in were excluded from library normalization or differential gene expression analysis. We include a gene for downstream analysis if it is differentially expressed at one or more time points between any 2 photoperiods. A total of 18 comparisons were made. This method was chosen as the time point of differential expression are of interest. For each comparison, using the “filterByExpr” function in edgeR only genes with at least 10 read counts in at least 3 libraries were kept for analysis. Read counts were normalized to trimmed mean of M-values for all subsequent analyses. Differential expression was defined at *p* < 0.2 for initial filtering or FDR < 0.05 for assessment of false discovery (Benjamini–Hochberg correction). Result is summarized in **S3 Data**.

### Daily expression integral calculation

The DEI, i.e., total expression of a gene across a 24-h day, was estimated with the area under the curve of the time course. First, the first data point at ZT 00 h was duplicated to extend the time course to ZT 24 h. Next, for each photoperiod, the area under the 24 h-curve was estimated using the trapezoid rule with the function “auc(method = ‘t’, design = ‘ssd’)” from the PK package to account for the serial sampling [63]. This generates 3 DEI values for each gene: DEI_EQ_, DEI_LD_, DEI_SD_. For easy visualization of the relative DEI of each gene in Fig 1B, the relative ratio of the exponentiated DEI, (DEI_EQ_)^4^, (DEI_LD_)^4^, and (DEI_SD_)^4^, was plotted as a stacked bar chart. The DEI ratio between 2 photoperiods (DEI_EQ_:DEI_LD_, DEI_EQ_:DEI_SD_, or DEI_SD_:DEI_LD_) was used for GSEA analysis (see below) and phenylpropanoid pathway analysis in **Fig 4**.

### Expression pattern analysis

All photoperiodic genes identified in the differential gene expression analysis were clustered using the APCluster R package [37]. First, Pearson’s correlation was chosen to measure the similarity in expression pattern across all 3 photoperiods. Next, clustering was performed with affinity propagation (similarity quantile = 0.5), wherein for each cluster, an exemplar gene that is most similar to all other genes according to a similarity score is selected. The resulted clusters were then merged with agglomerative clustering of the exemplars, yielding a dendrogram. A similarity cutoff of Pearson’s correlation = 0.82 was used to yield 14 major gene clusters. Detection of smaller clusters within the hierarchical clustering was performed with the DynamicTreeCut R package using the hybrid method with the deep split level set to 2 and 3. Expression patterns were plotted with ComplexHeatmap [64] and ggplot2 [65].

### Functional annotation analysis

Analysis was performed for gene groups defined at *p* < 0.2. All curated gene sets for *Arabidopsis thaliana* were downloaded from the “Plant Gene Set Enrichment Analysis Toolkit” online database [66]. GSEA of GO and KEGG terms was performed with the “gseGO” and “gseKEGG” function from the R package clusterProfiler [67]. For GO term GSEA only gene sets with a minimum size of 20 genes under the “biological process” categories were used. For GO and KEGG term enrichment analysis, the clusterProfiler function “enrichGO” was used and only gene sets with 10 to 500 genes were tested for enrichment. GO and KEGG terms that were enriched with a statistical level of false discovery rate < 0.01 are reported (Benjamini–Hochberg procedure).

### Motif enrichment and discovery

Analysis was performed for gene groups defined at *p* < 0.2. HOMER [68] was used to perform both enrichment of known motifs in CIS-BP and de novo motif discovery in gene promoters, defined as sequence from 1,500 bp upstream to 500 downstream of transcription start site in the TAIR10 gene annotation. CIS-BP motifs were downloaded from http://cisbp.ccbr.utoronto.ca/ and converted to HOMER format using the R package “universalmotif,” [69] and a mapping threshold of 8 was used to perform enrichment test. For de novo motif discovery default parameters were used. Motifs that were enriched with a statistical significance level of false discovery rate < 0.01 are reported. False discovery rate (Benjamini–Hochberg procedure) was calculated using the R function p.adjust(method = “fdr”) on the *p*-values reported by HOMER.

### Phase analysis

The function “meta2d” with default parameters from the R package MetaCycle was used to calculate phase of gene expression [70]. According to the method selection guidelines described by the authors, phase was estimated from the combined result of JTK and LS analyses (**S8 Data**). The ggplot2 function “geom_violin” was used to generate the violin plots in S7 Fig [65]. A Gaussian density kernel with 1.5 bandwidth was used.

### Analysis of enrichment of CO and PIF pathway genes in clusters

Hypergeometric test implemented by the “enrichr” function of the R package ClusterProfiler was used to calculate the enrichment of CO- and PIF-regulated genes in clusters with at least 100 genes at *p* < 0.2 [67].

### qRT-PCR

TRIzol reagent (Thermo Fisher, cat. # 15596026) was used to extract RNA from *Arabidopsis* seedlings according to manufacturer’s instruction. The RNA was treated with DNase (QIAGEN, cat. # 79254), and 800 nanograms of RNA were used for reverse-transcription using iScript Reverse Transcription Supermix for RT-qPCR (Bio-Rad, cat. # 1708841). qRT-PCR was performed with iTaq Universal SYBR Orange Supermix (Bio-Rad, cat. # 1725121). *AT4G05320* (*UBQ10*) was used as internal control gene. ΔΔCT method is used to calculate relative expression. Relative expression was calculated from 3 biological replicates. The primers are listed in S1 Table.

### LC-MS analysis of secondary metabolites

At ZT 4 of the 14th day post-germination, flavonols and anthocyanins were extracted from 150 mg of homogenized, flash-frozen whole seedlings in 750 μl of methanol:water:acetic acid (9:10:1 v/v). Cell debris was removed by centrifugation for 10 min at 14,000 g. The supernatants were transferred into new conical tubes and centrifuged again. Mass spectrometric measurements were performed with a Shimadzu Scientific Instruments QToF 9030 LC-MS system, equipped with a Nexera LC-40D xs UHPLC, consisting of a CBM-40 Lite system controller, a DGU-405 Degasser Unit, 2 LC-40D XS UHPLC pumps, a SIL-40C XS autosampler and a Column Oven CTO-40S. The samples were held at 4 deg C in the autosampler compartment. UV data was collected with a Shimadzu Nexera HPLC/UHPLC Photodiode Array Detector SPD M-40 in the range of 190 to 800 nm, and 10 μl of each sample were injected into a sample loop and separated on a Shim-pack Scepter C18-120, 1.9 μm, 2.1 × 100 mm Column (Shimadzu), equilibrated at 40 deg C in a column oven. A binary gradient was used with Solvent A (water, HPLC grade Chromasolv, with 0.1% formic acid) and Solvent B (acetonitrile, HPLC grade Chromasolv, with 0.1% formic acid). Flow was held constant at 0.3000 mL/min and the composition of the eluent was changed according to the following gradient:

0 to 2 min, held at 95% A, 5% B

2 to 10 min, change to 2% A, 98% B

10 to 18 min, held at 2% A, 98% B

18 to 18.01 min, change to 95% A, 5% B

18.01 to 20 min, held at 95% A, 5% B

Mass spectra were subsequently recorded with the quadrupole time-of-flight (QToF) 9030 mass spectrometer in the range from 100 to 2,000 m/z in negative ion mode (event time 0.1 s with 194 pulser injections) with subsequent data dependent MS/MS acquisition (DDA) for all ions in the range from 100 to 2,000 m/z with a collision energy of 35 +/− 17 internal units (event time 0.1 s with 194 pulser injections). The ionization source was run in “ESI” mode, with the electrospray needle held at +4.5 kV. Nebulizer Gas was at 2 L/min, Heating Gas Flow at 10 L/min, and the Interface at 300 deg C. Dry Gas was at 10 L/min, the Desolvation Line at 250 deg C, and the heating block at 400 deg C. Measurements and data post-processing based on accurate masses of the most abundant isotope (+/− 20 ppm) were performed with LabSolutions 5.97 Realtime Analysis and PostRun. Integrated peak areas representing mass spectral ion counts were normalized to the sample dry weight.

### ABSL quantification

Samples were collected at ZT 4 of the 14th day post-germination. Percent acetyl bromide soluble lignin (%ABSL) was quantified following a previously described protocol [71]. One gram of fresh weight seedling samples from plants grown as described was frozen in liquid nitrogen and ground using a Retsch MM400. Samples were then washed in 70% ethanol, chloroform/methanol (1:1 v/v), and acetone. Starch was removed from the samples via suspension in 0.1 M sodium acetate buffer (pH 5.0), heating for 20 min at 80°C, and addition of 35 μl amylase (MP Biomedicals, LLC, Lot # SR01157) and 17 μl pullulanase (Sigma-Alrich, Lot # SLCC1055). Samples were left shaking overnight at 37°C before termination of digestion. The samples were washed using water and acetone, dried, and then ground to a powder to facilitate accurate mass measurements for lignin quantification. Between 1 and 1.5 mg of cell wall material was suspended in 100 μl acetyl bromide solution (25% v/v acetyl bromide in glacial acetic acid) and heated at 50°C for 3 h with vortexing every 15 min during the third hour. Samples were cooled to room temperature before addition of 400 μl of 2 M sodium hydroxide, 70 μl of 0.5 M hydroxylamine hydrochloride, and 1430 μl of glacial acetic acid, and 200 μl of the resulting solution was used to measure absorbance at 280 nm and calculate %ABSL using Beer’s law with a coefficient of 15.69 for *Arabidopsis thaliana*.

## Supporting information

S1 Fig The experimental design for constant photoperiod and shifted photoperiod growth conditions. Gray and dark bars represent light and dark periods, respectively. The first time point is zeitgeber time hour 0 (ZT00). Zeitgeber time is equal to the number of hours from dawn. The shifted photoperiod design is identical to that illustrated in **Fig 1A**. However, for better visual comparison, the EQ time course of the shifted photoperiod condition is not plotted in **S8 Fig**.
(EPS)

S2 Fig Expression of selected marker genes in constant photoperiod versus that in shifted photoperiod. Gene expression in constant photoperiod is detected by qRT-PCR with *UBQ10* as reference gene. Gene expression in the shifted photoperiod is detected via RNA-sequencing and is presented in the unit of reads per kilobase of transcript per million reads (RPKM), with the exception of *FT* which was detected by qRT-PCR with *UBQ10* as reference gene. Blue: SD expression; red: LD expression. Expression mean is shown and error bars indicate standard deviation in both qRT-PCR and RNA-seq (*n* = 3). qRT-PCR data is available in **S1 Data**. Gene read counts are available in **S3 Data**.
(EPS)

S3 Fig Differentially expressed genes between time points and photoperiods. **(A)** Upset plot of differentially expressed (DE) genes in each time point at *p* < 0.20. For simplicity, only groups with at least 100 genes are plotted. **(B)** Upset plot of DE genes in each photoperiod at *p* < 0.2. **(C)** Upset plot of DE genes in each time point at FDR < 0.05. **(D)** Upset plot of DE genes in each photoperiod at FDR < 0.05. Data is available in **S4 Data**.
(EPS)

S4 Fig Gene exemplars from the 14 major clusters selected from affinity propagation. *n* refers to the number of genes in cluster. Blue: SD expression; orange: EQ expression; red: LD expression. Gene read counts are available in **S3 Data**.
(EPS)

S5 Fig Gene set enrichment analysis (GSEA) with rDEI_LD:EQ_ and rDEI_EQ:SD_ as ranking metric. **(A)** Ridgeplot showing the distribution of the leading edge genes of top KEGG pathway terms of GSEA using DEI ratio between LD and EQ as ranking metric. **(B)** Ridgeplot showing the distribution of the leading edge genes of top gene ontology and KEGG pathway terms of GSEA using DEI ratio between SD and EQ as ranking metric, and *p*-value was adjusted using the Benjamini–Hochberg procedure. Only the top 10 terms ordered by absolute normalized enrichment score (NES) that pass the threshold of adjusted *p*-value < 0.2 are shown. Data is available in **S6 Data**.
(EPS)

S6 Fig Multidimensional scaling plot of sample triplicates. Numbers represent the ZT hour of sample collection. Color indicates photoperiod condition. Data is available in **S4 Data**.
(EPS)

S7 Fig Gene exemplars from subgroups where EQ-induced peaks were observed. Blue: SD expression; orange: EQ expression; red: LD expression. Gene read counts are available in **S3 Data**.
(EPS)

S8 Fig Median Z-score expression values of major subgroups generated by affinity propagation. Error bars indicate 25th and 75th quantile of Z-score expression values. Blue: SD expression; orange: EQ expression; red: LD expression. Gene read counts are available in **S3 Data**.
(EPS)

S9 Fig Violin plots of phase distribution of genes in clusters 3N, 4I, 4J, and 11J. Inner box plot in gray indicates 5th, 25th, 50th, 75th, and 95th quantile of the distribution. Blue: SD; orange: EQ; red: LD. Data is available in **S9 Data**.
(EPS)

S10 Fig Simplified phenylpropanoid biosynthesis pathway with gene subgroup membership. Box labeling corresponds to biosynthetic enzyme names. Genes with no subgroup labels or shading did not display photoperiodic expression patterns or consist of multiple homologs that do not show consistent expression patterns. Data is available in **S10 Data**.
(EPS)

S11 Fig Expression of all detected phenylpropanoid biosynthetic genes listed in Fig 4B in reads per kilobase of transcript per million reads (RPKM). Expression mean is shown and error bars indicate standard deviation (*n* = 3). Blue: SD expression; orange: EQ expression; red: LD expression. Gene read counts are available in **S3 Data**.
(EPS)

S12 Fig Schematic model of the control of photoperiodic gene expression and downstream biological processes. (A) In SD, genes are induced in 3 major ways: (A1) an unknown mechanism increases expression amplitude of a day-phased peak, up-regulating genes involved in hypoxia response and amino acid metabolism; (A2) MDLM likely induces gene expression after the earlier dusk in SD through the AP2/ERF-family TFs, in turn up-regulating genes involved in processes like hypoxia response, amino acid catabolism, and response to darkness; and (A3) TFs binding to G-box and AP2/ERF TFBS trigger gene induction in darkness, leading to up-regulation of genes involved in various processes. (B) In LD, genes are induced in 4 major ways: (B1) MDLM likely induces an expression peak in the latter part of daytime via G-box binding TFs, causing an up-regulation of genes involved in processes such as desiccation response; (B2) an unknown mechanism drives the expression of genes under light, leading to an up-regulation of genes involved in glucosinolate metabolism; (B3) G-box binding TFs induce higher expression in the latter part of daytime, in a manner similar to (B1), causing up-regulation of photosynthesis genes; and (B4) an unknown mechanism causes an expression peak in the dark, up-regulating genes involved in various processes. (C) Photoperiod controls expression of circadian clock- and rhythmic process-related genes in 4 major ways: (C1) evening element-containing genes display an SD-specific mid-day peak, thus also causing SD-induction; (C2) G-box binding TFs trigger the increase in magnitude of a dawn-phased peak in SD; in (C3) and (C4), evening element-containing genes with a mid-day phase show a phase delay with lengthening photoperiod; the SD phase may be restricted to light in (C3) or extend to the dark in (C4). (D) In LD, ribosomal genes containing the TFBS for TRB-related TFs display an expression trough in the middle of the daytime period. Red lines, orange lines, and blue lines indicate expression in LD, EQ, and SD photoperiod, respectively.
(EPS)

S1 Data Real-time quantitative PCR data of selected genes in constant and shifted photoperiods. (XLSX)

S2 Data Summary statistics of RNA-sequencing libraries and mapping. (XLSX)

S3 Data Read count of detectable genes in each RNA-sequencing library. (XLSX)

S4 Data Results of edgeR differential expression analysis and principal component analysis. (XLSX)

S5 Data Cluster membership of genes and gene daily expression integral. (XLSX)

S6 Data GSEA results and GO enrichment data of gene subgroups. (XLSX)

S7 Data Enrichment analysis of CO- and PIF-regulated genes in gene subgroups. (XLSX)

S8 Data *cis*-element enrichment analysis of gene subgroups by HOMER. (XLSX)

S9 Data LD, EQ, and SD phase calculation using the MetaCycle package. (XLSX)

S10 Data Catalog of phenylpropanoid biosynthesis genes. (XLSX)

S11 Data LC-MS ion count quantification of phenylpropanoid-related compounds and acetyl bromide soluble lignin quantification in LD and SD. (XLSX)

S1 Table Primers used in qRT-PCR analysis. (XLSX)

## Abbreviations

ABSL: acetyl bromide soluble lignin
DEI: daily expression integral
EQ: equinox
GO: Gene Ontology
GSEA: gene set enrichment analysis
KEGG: Kyoto Encyclopedia of Genes and Genomes
LC-MS: liquid chromatography–mass spectrometry
LD: long day
MDLM: metabolic daylength measurement
PIF: phytochrome-interacting factor
QToF: quadrupole time-of-flight
rDEI: relative daily expression integral
SD: short day
TF: transcription factor
TFBS: transcription factor binding site
